# Contemporary Theory and Research

**Published:** 1893-07-22

**Authors:** 


### Contemporary Theory and Research.

### PROGRESS IN JAPAN.

The malady known among the Japanese as "kak'-ké," and in Europe as béribéri, has been completely extinguished in the Japanese navy solely by means of a change and improvement in the diet of the sailors. In 1884 there were 718 cases; in 1885 and 1886 they diminished to an extraordinary extent, and by 1889 not a single case remained. The improvement in the rations has, moreover, worked so remarkable a change in the condition of the sailors that in the course of five years their chances of illness sank from 3·65 in a year to ·39.

### A NEW SUBSTITUTE FOR SUGAR.

A new substance known as dulcine (para-phenetolcarbomide) is recommended in place of saccharine for sweetening purposes in cases where sugar may not be taken. Herr Rossel, who has studied the physiological action of dulcine, states that it is absolutely without harmful properties. He has administered it to rabbits and dogs in doses of about 30 grains a day, and this dose, which corresponds to about a pound of sugar, can be continued for months without affecting the digestive organs or producing any disorder or inconvenience. Herr Eweld has tested it on some of his patients with perfectly satisfactory results.

### SCENTS AS ANTISEPTICS.

M. Lucas Championnière has presented to the Académie de Médecine an account of some endeavours to extend the use of scents having antiseptic properties in the sick-room. "During some years I have tried to suppress from my practice powerfully toxic antiseptics and those diffusing an objectionable odour. Starting from the laboratory researches of M. Chamberland, I have studied the antiseptic properties of scents, and specially of the extract of cinnamon, which has long been known for its disinfecting qualities. According to M. Chamberland, indeed, it is an even more powerful antiseptic than sublimate. At the commencement I was much embarrassed by the fact that the extract had an irritant action, but I overcame the difficulty by dissolving the essence in restinol, a very gentle antiseptic which is obtained from resin, and I succeeded in obtaining a very useful preparation called by M. André cinamol. The results obtained in the treatment of wounds have been very remarkable. I have also tried other essences: essence of marjoram of geranium, of verveine, &c. These various extracts have a better scent, and are less irritant. A mixture also of these different extracts can be utilised, and an antiseptic obtained which is at once very gentle and very active."

### HOP-PICKERS' OPHTHALMIA.

Dr. Percy Adams has been investigating an affection of the eyes which specially attacks hop-pickers, and appears to be the consequence of rubbing the eyes with the hop-stained hands. In its mild form it is a simple conjunctivitis, but it sometimes involves deeper structures, and keratitis and hypopyon result. It does not specially attack those who are exposed to unsanitary conditions like the foreign pickers who sleep in barns or tents, but is fairly equally distributed among all the pickers, women and children being more readily affected than men. It seems certain that the ophthalmia is due to a mechanical cause, *viz.* to the thorn-like hairy processes which cover the tracts of the hop catkins, and which are very sharp. Alcoholic extract of hops, evaporated to dryness and introduced into the eyes, produced no effect beyond a little smarting.

### THE BACILLUS ANTHRACIS.

It appears from the "Annales de l'Institut Pasteur" that M. Diatroptoff has discovered the bacillus anthracis in the mud at the bottom of a well. An epidemic of splenic fever having broken out among some sheep on a farm in the South of Russia, the source of infection was traced to a well. The water was bacteriologically examined without result, but in the mud at the bottom M. Diatroptoff found an organism which, on inoculation into other animals, proved to be the bacillus anthracis. After the well was closed no more cases were reported. It is certain that in some way the germs of anthrax had gained access to the well, and this, as a writer in *Nature* observes, opens up a serious consideration of the possibility of the spread of infection through drinking water, and the question as to whether, in view of the contamination of the surrounding soil by the burial of infected animals, it might not be wise to insist on their cremation. It may be mentioned that an elaborate report has lately been issued to the Water Research Committee of the Royal Society entitled "The Vitality and Virulence of Bacillus Anthracis in Potable Waters," prepared by Percy Frankland and M. Ward.

